# Finite element analysis and structure optimization of a gantry-type high-precision machine tool

**DOI:** 10.1038/s41598-023-40214-5

**Published:** 2023-08-10

**Authors:** Tzu-Chi Chan, Aman Ullah, Bedanta Roy, Shinn-Liang Chang

**Affiliations:** 1 Department of Mechanical and Computer-Aided Engineering, National Formosa University, Yunlin County, 632 Taiwan, R.O.C.; 2 Department of Power Mechanical Engineering, National Formosa University, Yunlin County, 632 Taiwan, R.O.C.

**Keywords:** Mechanical engineering, Engineering

## Abstract

The high-precision machine tool’s dynamic, static, and rigid nature directly affects the machining efficiency and surface quality. Static and dynamic analyses are essential for the design and improvement of precision machine to ensure good tool performance under difficult and demanding machining conditions. In this study, the performance of a high-precision machine tool was analyzed using its virtual model created using CAD. Static and model analysis using ANSYS Workbench software was conducted to establish the tool's static deformation and static stiffness. Furthermore, the static and dynamic characteristics of the tool were explored using a finite element modeling approach to study their performance. In particular, the structure, static force, modal, frequency spectrum, and topology optimization of machine tools were primarily analyzed. Using model analysis, we found the first four different frequencies (22.5, 28.9, 40.6, and 47.4 Hz) and vibration type, which suggested of a weak link. Further static structural analysis revealed that the deformation of the spindle was 67.26 μm. An experimental static rigidity analysis was performed, and the experimental deformation values of the tool and spindle were obtained. The static and dynamic characteristics, as well as the accuracy and efficiency of the finite element model, were verified by comparing the data with the finite element analysis (FEA) results. Subsequently, we modified the settings and analysis model to ensure that the analysis results were consistent with the experimental findings. The error between the two results was within 1.56%. For an applied load of 5000 N on the spindle nose, the tool nose transient response was 0.5 s based on transient analysis. Under the condition that the structural deformation is as constant as possible, the lightweight structure may achieve the minimum weight and enhance the natural frequency; thus, the ideal structure will be obtained, and finite element analysis will then be performed. The optimal conditions for topology optimization include a lightweight structure, reduced structural deformation, and increased natural frequency of the structure. The developed method improves structural optimization, increases the ability of the product to be manufactured, and offers designers a variety of price-effective options.

## Introduction

Market competition is constantly encouraging precision machine tool manufacturers to improve productivity while lowering machine prices, by establishing a downward trend in machine tool energy consumption. Therefore, lightweight precision machine tool structure design is now widely used for energy efficiency. However, the capacity to reach the top limits of servo drivers is an important factor in producing efficient precision machine tools. Currently, mechanical computer-aided design (CAD) is mostly used in designing precision machine tools because it is not necessarily required to physically build the model, assemble the casting, or identify the weakest area of the structure; thus, it significantly reduces the cost of development and shortens the development time. In recent years, in response to the booming production of defense, semiconductor, and 3C industries, the processing speed and precision of components have increased along with the demand for hard and brittle materials. To fulfill the dynamic and static rigidity of the high-precision machine tool, improving the casting will have a considerable effect on achieving improved performance and a fair reduction on production costs. However, the casting of the machine tool is mainly designed through people's long-term experience, which may not meet the requirements; furthermore, each design must be actually produced for testing, which is a waste of time and money. Various research works on industrial machine tool design methodologies^1–4^, and a variety of commercially accessible precision machine tools are available^5,6^. However, it is crucial for manufacturing companies to pursue intelligent manufacturing by creating intelligent precision machine tools that use an in-depth fusion of cyber-physical systems to increase product quality and throughput while reducing costs^7,8^.

**Figure 1:**
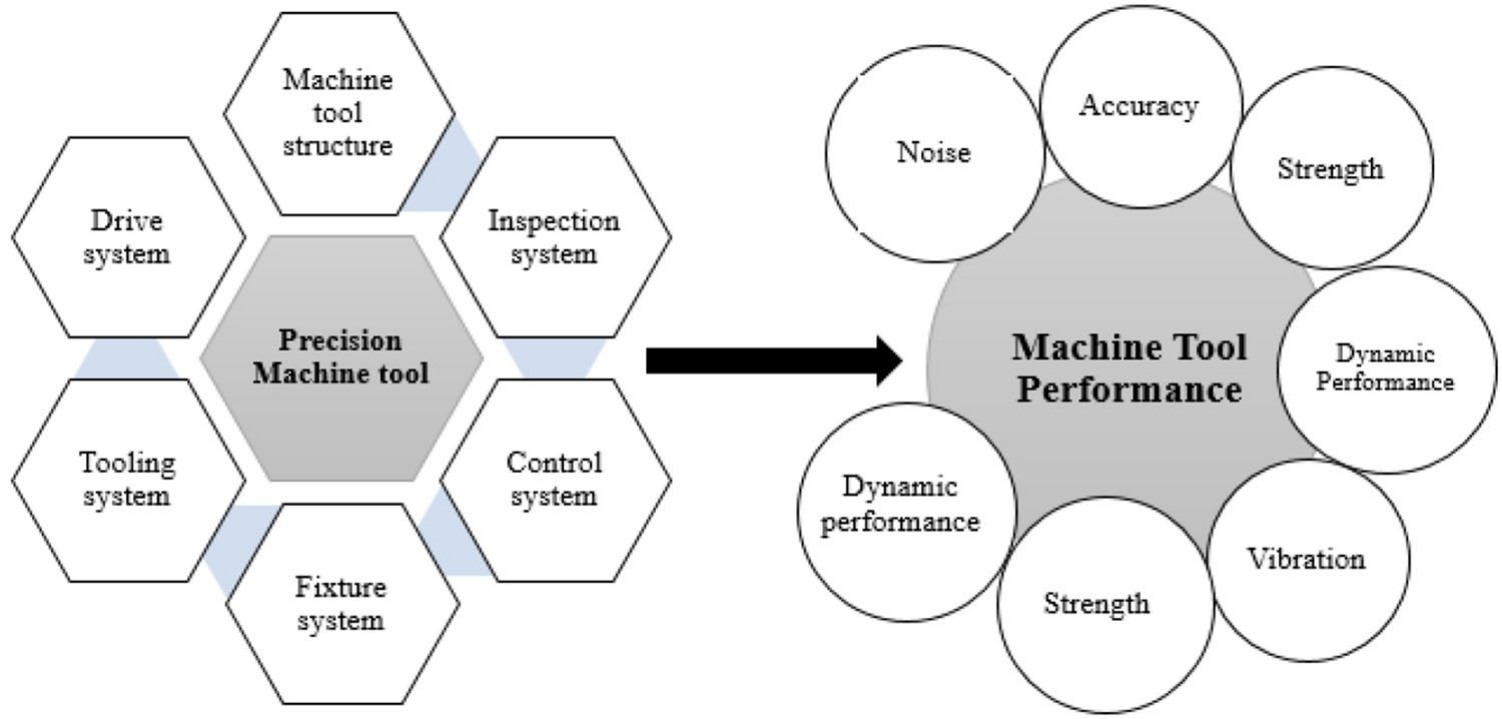
Machine tool performance and components.

Figure 1 shows the components of a basic machine tool as well as the essential selection for evaluating different types of tool performance. Because different companies use different manufacturing methods and setups to obtain the required machine performance, it is necessary to design machine tool subsystems as stated. The results of the assessments were common in the following aspects:

- Precision
- Kinematics
- Stability
- Motion
- Durability
- Temperature
- Sound
- Vibration

Individually and collectively, the machine performances shown in Fig. 1 reflect the tool–workpiece loop in terms of stiffness, thermal stability, statics, and dynamics. In the following sections, the tool–workpiece loops are the main topic of discussion, with a possible emphasis on machine dynamics. Machine tools are important in the industry, and there have been significant improvements in productivity, accuracy, and durability in recent years. The desired outcome may be attained using a literature review of previous knowledge based on the design of a Computer Numerical Control (CNC) gantry machine. Hyun Surk Kim et al.^9^ studied a comprehensive modeling approach for linear motion guides and ball screws utilizing the three-dimensional finite-element method was introduced for use in the grinding machine's stiffness calculations. Schellekens and Rosielle^10^ investigated the basis of the planned experiment, and the effect of the sample temperature and holding duration during diffusional surface alloying on the hardened layer thickness was examined. Choosing the right materials for a precision machine tool structure affects the machine performance in numerous ways, including temporal constancy, particular static stiffness, simplicity of manufacturing, and cost. Lan^11^ explored the current CNC turning business, where surface roughness is a key factor. They proposed an orthogonal array-based parametric deduction optimization technique and made a profoundly insightful contribution to CNC turning, wherein a fuzzy approach to surface roughness was used. Tekeste et al.^12^ analyzed gears using finite element analysis software and found that the spur gear root was exposed to the greatest stress, and the gear root position was the position where the gear was easily damaged. Using CATIA and ANSYS software, an involute spur gear was modeled to investigate the contact stress under various operating conditions. Xu et al.^13^ reported that, in previous gantry designs, the first natural frequencies were rarely higher than 100 Hz, and the gantry frequency of a machine should be greater than 140 Hz. However, the normal frequency of milling machines is below 150 Hz. Because vibration degrades product quality, manufacturers aimed to adjust the methods to create the most stable machining gantry. Yongqing et al.^14^ investigated the static and dynamic properties and found that under maximum cutting force and gravitational load, the stiffness of the crossbeam and the tip displacement were transformed by beam deformation. Miroslav Pastor et al.^15^ used Finite Element Method (FEM) to properly estimate crane girders with an arched span such as a bridge. This approach provided several deflection calculation strategies for bridge-type crane girders and allowed for the inference of the calculation formula of webs baiting arch degree curves prior to production. Zhao et al.^16^ enhanced the structural properties of a crossbeam for an MC-6000 gantry model center. They used a bionic technique inspired by the ribs of a large waterlily petal and cactus stem structure. The effectiveness of the bionic technique was demonstrated by a 16.22% reduction in static deformation and 3.31% reduction in weight. Several researchers have applied these strategies effectively during the design phase^17,18^. The design of machine tools frequently involves competing objectives, such as dynamic, static, weight, and cost-effectiveness. Various techniques for multi-criteria decision-making have been suggested, including AHP, ANP, TOPSIS, ELECTRE, PROMETHEE, GRA, and PEG-MCDM. All aforementioned studies have focused on improving the dynamics and quality of the horizontal beam of the gantry tool, which is caused by the high rigidity of its fixed wall. The results revealed that the proposed design procedure for a gantry with performance-oriented moving columns was effective. Mayr et al.^19^ studied the static analysis software that must be utilized before manufacturing to investigate the precision of the static behavior of the machine tool structure to discover various machining errors. Maia^20^ investigated the problem of vibration-generated resonance by analyzing a structure with machine characteristics. Budak and Altintas^21^ used measurements of stiffness and modal analysis and the dynamic and static investigation of the machine tool components used in machining systems. Eman and Kim^22^ proposed a method that utilizes the representation of measurable responses as parameters for the modal analysis of precision machine tool structures. Their proposed method was easily applicable to computer-based analyses of the structural dynamics of precision machining tools. Pedrammehr et al.^23^ described the milling machine dynamics and modal parameters. For this purpose, CATIA supplies the milling machine with a structural CAD model. The ANSYS Workbench FEM modal analysis determines the precision of the machine tool’s structural natural frequencies and mode shapes. Guo et al.^24^ suggested a method for optimizing the dynamic performance of a grinding machine spindle that uses a dynamical frequency-domain response. Therefore, the dynamic rigidity of the grinding machine should be the primary focus of the process to optimize the dynamic functionality of the machine. Cao et al.^25^ studied the purpose of assisting industrial engineers in acquiring a trustworthy model that can precisely describe the dynamic features of machine-tool spindle systems. A frequency response function-based (FRFs) iterative approach was used to update the FE model. Huynh and Altintas^26^ introduced a simulation of the multibody dynamics of five-axis machine tools using a system modeling approach. The proposed model was capable of accurately predicting coupled rigid-body motion and machine vibration using limited computational resources. Tong et al.^27^ optimized many objectives used when designing a spindle bearing system, including the natural frequency, total friction torque, bearing position, static stiffness, and bearing preload; one of these was chosen to serve as the design variable. Kim^28^ performed a case study that investigated and assessed the dynamic stiffness of a 5-axis ram-head type multi-tasking machine tool. Chan et al.^29^ examined the machine tool performance evaluation of the structure and verified it by testing. In conclusion, the quality of the machine used for processing determines the performance of the experiment. Fedorynenko et al.^30^ experimentally investigated spindle dynamics. The modeling results and actual spindle dynamics were in good agreement. Simon^31^ studied the structural frequency response functions, which usually have several peaks. The FRF spectrum resonance of the structure is key to modal analysis. The structural behavior can be examined by detecting and evaluating all spectrum resonances and modes. Zhao et al.^32^ created an innovative approach for regulating topology when performing structural optimization. The efficiency of the proposed method was demonstrated using standard numerical examples, which include the minimization of compliance for unclamped, MMB, and 3D cantilever beams, all within the context of various structural constraints. The structural and dynamic qualities of the crossbeam affect the accuracy and stiffness of the gantry precision machine tool. Yaguang Wang et al.^33^ proposed that multi-material systems with graded interfaces can effectively describe and optimize the width of the interfacial region and graded interfaces by employing a topology optimization method to reduce the maximum stress. Ee et al.^34^ This work shows a FE model for studying the formation of residual stress in 2-D machining that is meant to be more realistic. Liu et al.^35^ studied a mechanical device utilizing a gantry design of a crossbeam structure involving decision-making across multiple dimensions (levels), indices (measures), and schemes. As a result, studies aimed at perfecting the structural design of the crossbeam for gantry machine tools are increasing. Han et al.^36^ indicated that a multi-material construction including a graded interface can be defined and optimized in an effective manner based on the study findings. It is possible to accurately regulate and determine both the width of the interfacial zone and the properties of the graded interface. Chan et al.^37^ the transient response analysis was performed and the impact of the axial cutting force during machining was examined to determine the impact of vibrations on processing quality. In addition, a harmonic analysis of the machine was carried out. Finally, we minimized the machine tool's overall mass by optimizing its structural design. Yiming Zhang et al.^38^ Used method based on the theory of static deformation is used to find the elastic rocking modal frequency of the disk-shaped rotor in MSCMG. The effect of the structure's factors on the rotor's rocking frequency is studied. Lastly, the rotor is optimized from many different points of view.

**Figure 2:**
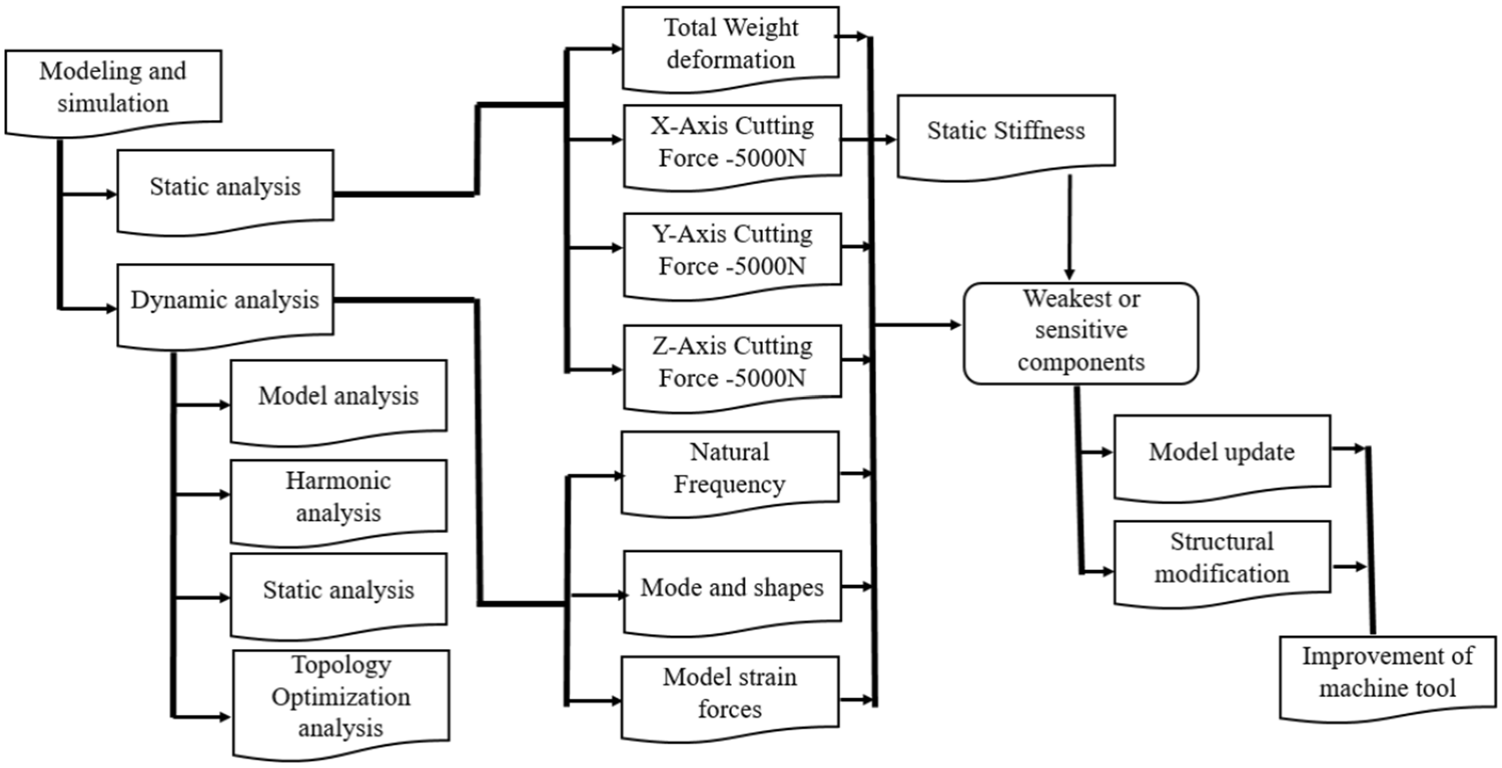
Flowchart of the machine tool analysis.

Techniques for analysis and simulation include modeling of important components, system modeling, static analysis, and dynamic analysis. FEM is a commonly used analytical and simulation technique. The results of the analysis, budget, and cost projections with accompanying inaccuracy were used to determine conformity according to the requirements of the machine. The results also help in identifying the weakest structural components of a precision machine tool and provide information on structural improvements, thereby accelerating the decision-making process. The engineering environment for the development of precision machine tools should incorporate structural design, analyses, and experiments. Because there are numerous unknowns in a pure analysis and simulation process, particularly when dealing with a completely built novel design configuration, experimental dynamic testing was initially utilized to support dynamic simulation. Present Investigation revolves gantry-type high-precision machine tool which during operational experimentation displayed moderate material and structural strength. Using finite element method, high fidelity, topology optimization, iterative process was implicated on the shape of machine to improve material and structural strength. Additionally, a harmonic analysis was done before optimization. The experimental static stiffness of the tool and spindle was analyzed, and the experimental deformation values of the bed and tool were determined. By comparing the data with the results of the FEA, the static and dynamic characteristics of the finite element model, as well as its accuracy and efficiency, were verified. Furthermore, we reduced the machine tool's overall weight by optimizing its structural design. The proposed technique can adequately represent the static and dynamic characteristics of large machine tools, as shown in Fig. 2. Consequently, model, structural, and simulation verification tests were performed using FEA, which has a considerable number of applications for structural analysis.

## Materials and methods

### Large machine tool design method based on simulation

**Figure 3:**
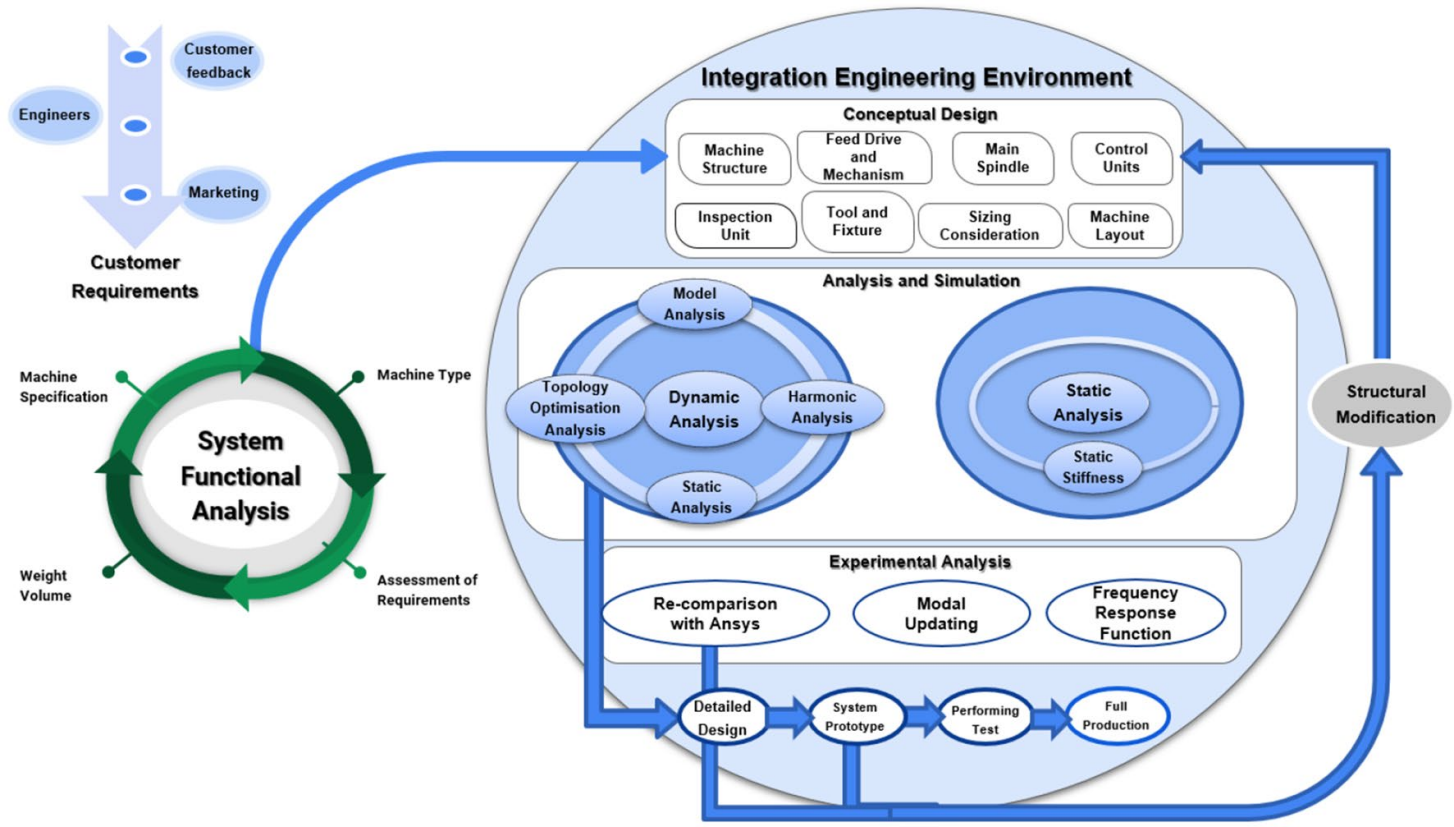
Precision machine tool design technique.

Overall, design is an ongoing, simultaneous, non-linear, multidisciplinary process that is constantly open to new practical concepts and enhancements. Factors such as design, kinematics, dynamics, power requirement, materials, detector and control mechanisms, safety devices, ergonomics, manufacturing, disassembly, quality management, transportation, operation, budget, and timetable may need to be included in the functional requirements of a precision machine tool. For the design to be more competitive and economical, the latest technology must be used. The final specifications are then set after a few iterations. The conceptual design that emerges from this is important for improving the design of precision machines. Precision machine tools include several different aspects, each of which contributes to the final product. As depicted in Fig. 3, the customer needs objectives, design development, analysis and simulation, experimental investigation, design specifications, design follow-up, and system functionality. Understanding and defining consumer needs and requirements begins product development. Market research, surveys, and consumer feedback are used to determine product features, functions, and performance. After identifying consumer needs, engineers and designers create conceptual ideas and prototypes that meet them. After then, analysis and simulation are used to test designs under different scenarios to ensure safety and quality. Experimental tests can verify the design's real-world performance. Design specifications establish the product's parameters and traits. Progress, changes, and compliance with specifications are monitored throughout the process. Finally, the product's functionality is tested to ensure it meets client needs and fulfills its purpose.

**Figure 4:**
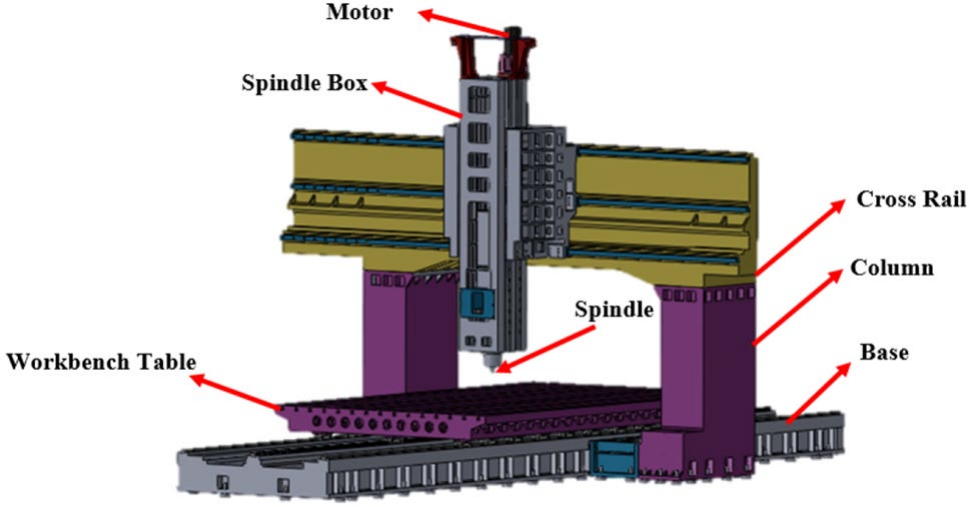
Design of multi-function giant sliding double column machine.

Using CAD to establish the model, the main parts include a moving table (X-axis), sliding cross rail (Y-axis), base (Y-axis), spindle box (Z-axis), saddle (Z-axis), and column (Z-axis). All these parts were made of cast iron, whereas other parts including motorized spindle, fixed sports, gearbox, motor, slider, and machine tool were made of structural steel. The work area of the machine table was 5000 mm, with a width of 2600 mm and a maximum table load of 25 tons. The spindle speed was 0–6000 rpm, in which the spindle was driven by a spindle motor, as listed in Table 1. The CAD file was introduced in the ANSYS environment and series of strength analysis were performed. Figure 4 shows the model used in this study.

**Table 1:** Machine model specifications ^39^.

Item		Unit	
Distance between columns		mm	3650
Table size	Width	mm	5000
	Length	mm	3000
Maximum table load		ton	22
Travel	X-axis	mm	5200
	Y-axis	mm	3600
	Z-axis	mm	1500
	W-axis	mm	1000
Spindle	Nose to table	mm	260–1360
	Ram size	mm	450 × 400 (opt. 550 × 550)
	Taper/Speed/Power	Rpm/kW	BBT 50–6000 rpm − 22/26 kW + 2 stage gearbox
Federate	Cutting	m/min	10
	X-axis	m/min	8
	Y/Z/W-axis	m/min	2/10/1
Accuracy		mm	Positioning ± 0.015/full travel ± 0.003; Slope of cross rail 0.01/1000 Positioning ± 0.015/full travel ± 0.003; Slope of cross rail 0.01/1000
ATC & tool magazine	Capacity/dia	mm	32 tools, max. dia Ø125 (Full tool)/Ø220 (Adjacent empty)
	Max. length/weight	mm/kg	400 mm/20 kg
	Tool selection		Random shortest direction/M24 P3.0–45°
Machine size	Length	m	11.2
	Width	m	6.8
	Height	m	5.9
Machine weight		ton	72

### Methodology

**Figure 5:**
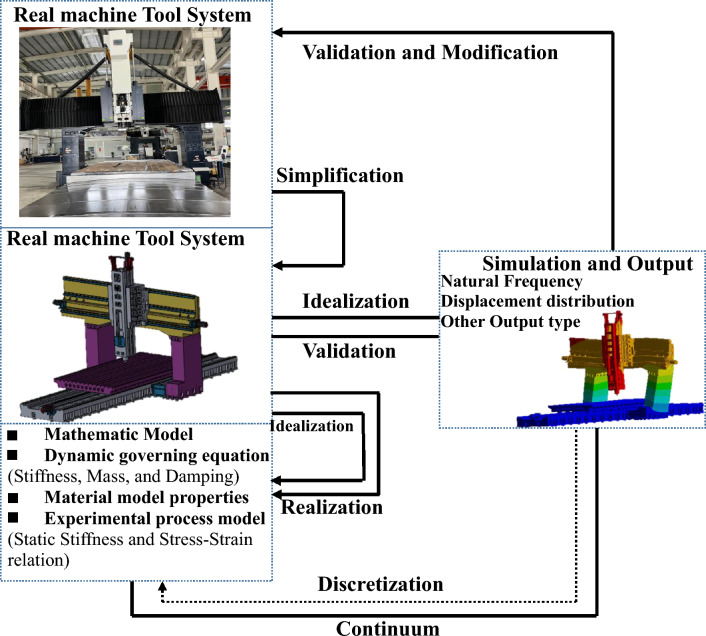
Generalized method of modeling and simulation.

In terms of machine design, modeling can be utilized to depict both the machine operations and tool structure. Consequently, the simulated model may create a link between the components and results, enabling a mathematical or graphical representation of the static or dynamic performance of the machine tool. The Numerical technique may help bridge the gap between the theoretical and practical models of the machine as well as the theoretical and practical aspects of its dynamic performance. The dynamic performance of a real machine tool system was simulated using this modeling and simulation approach, as shown in Fig. 5. Because it is challenging to create an accurate model of a machine's complex system, the ideal physical model is a simplified depiction of a real machine system. Some minor changes such as the motor, filling holes, chamfer, and filters were removed and have been ignored as a result of the simplification. To solve the physical model, a mathematical model was developed, which was derived inductively from first principles. Ideal physical models may be considered implementations of the mathematical model, and the mathematical model can be considered an idealization of the ideal physical model.

### Materials

The machine’s main parts include a sliding table, sliding cross rail, base, column, spindle box, and saddle, all of which are cast iron materials whose properties are listed in Table 2. Other parts are motorized spindles, fixed sports, gearboxes, motors, sliders, and machine tools made of structural steel, which have the properties listed in Table 3.

**Table 2:** FC300 material properties.

FC300	
Density (kg/m^3^)	7300
Young's modulus (Pa)	1.15E + 11
Poisson's ratio	0.25

**Table 3:** Structural steel material properties.

Structural steel	
Density (kg/m^3^)	7850
Young's modulus (Pa)	2E + 11
Poisson's ratio	0.3

### Finite element model

**Figure 6:**
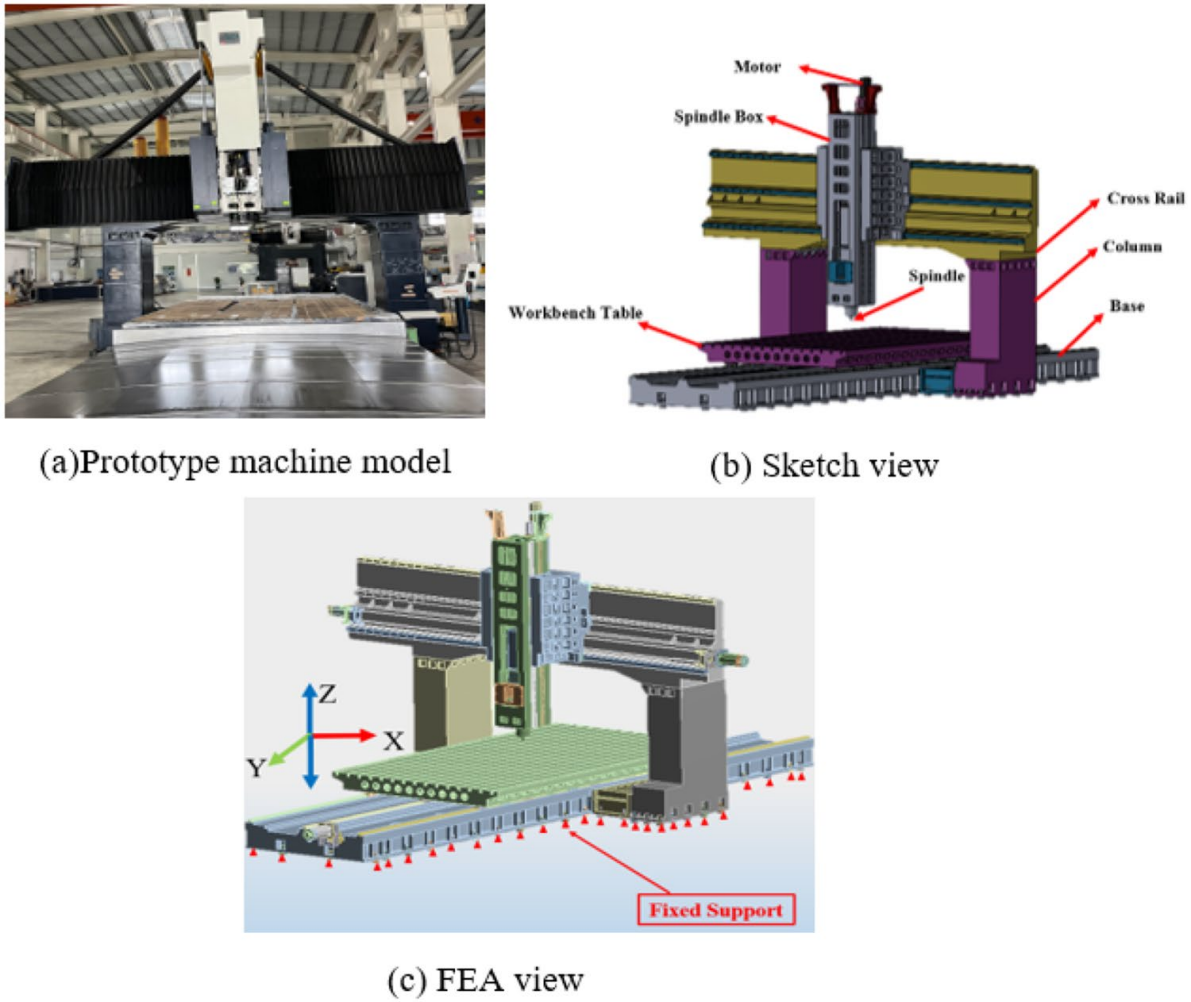
FEA structures of the large machine tool.

Improvements in the structure and essential parameters were achieved through extensive parameter analysis. Figure 6 shows the FEA of a high-precision machine tool with fixed support that uses solid elements for the simulation. To obtain a high-precision solution, high-precision machine tools parts such as the workbench, fixed supports, slider, and tool should be subdivided into a fine grid for analytical purposes. This requires considerable computing power and a long solution time.

CAD models of high-precision machine tools include many extra bodies, holes, and slots, which make it difficult to mesh them and increase the calculation accuracy. Before creating an FEA model, it is important to simplify the geometric solid model of a high-precision machine tool. Figure 6 shows a schematic of the high-precision machine tool system. Rather than using a simplified sharp edge, a new analytical expression of the friction coefficient at the tool-chip-work-piece interface was created, allowing more precise FE friction modeling. The unique analytical formula, FE simulation, and experimental verification of outside-diameter grooving with an uncoated carbide tool establish the mean friction coefficient m used in the FE model in the HSM^40^. In recent years, multiscale FEM has become a popular and useful model for analyzing the non-linear behavior of structures^41–45^ which has high modeling and solution efficiency. In multiscale FEM, a large number of solid elements are replaced by shell or beam elements. In addition, the relationships between solid, shell, and column elements are handled using appropriate modeling techniques. This ensures the accuracy of the solution, reduces the number of elements and nodes in global structures, speeds up the solving process, and obtains accurate partial structural stress characteristics.

## Mesh of model structure

### Mesh configuration settings

Verifying their influence on simulation fidelity in a 3D finite element model preceding meshing^46,47^ prevented an excessive number of fine grids and elements that would otherwise impair the calculation time for finite-element simulations.

**Figure 7:**
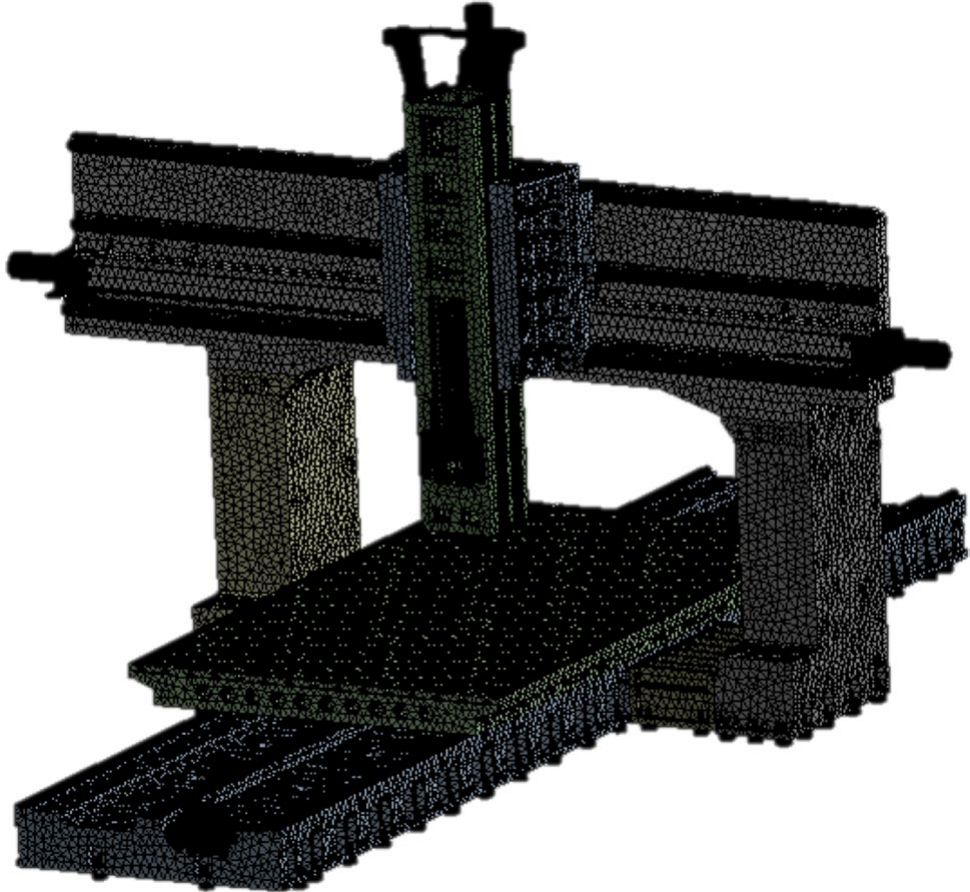
Mesh model.

The majority of the parts of the high-precision machine tool had irregular geometric shapes. This study meshed the grids by considering the tetrahedral element machine as a whole. The mesh was divided into two categories, as listed in Table 4. After meshing the number of nodes and elements are labeled in Table 5, and the final model is illustrated in Fig. 7.

**Table 4:** Mesh grid settings of the large machine tool.

Parts name	Mesh size, mm
Base, beam, column combination seat, z-axis	70
Workbench	70
Column, slide	70
Feet	20
Spindle, slide rail, slider, motor	20

**Table 5:** Mesh gird result.

Mesh average	0.54
Number of elements	1,499,510
Number of nodes	2,646,952

### Mesh generation and mesh independence study

**Figure 8:**
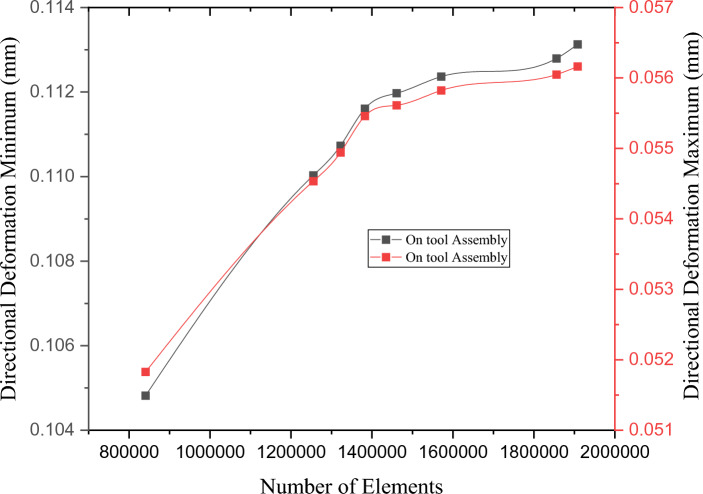
Maximum and minimum directional deformation (mm).

**Figure 9:**
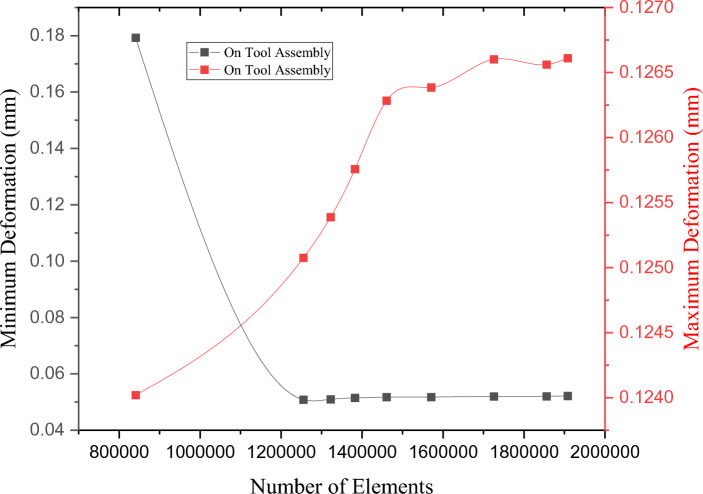
Maximum and minimum deformation (mm).

In general, finer finite element mesh can effectively capture a geometry's outlines and more “data points” on the geometry are available to provide a precise displacement and stress response. To determine the independence of a mesh, an analyst may use grid-independence research to ascertain the extent to which the outcomes depend on the mesh density. One method for accomplishing this is to use the strategy outlined below. There are other methods for employing mesh quality metrics to directly check the mesh quality, as stated previously. Three distinct cases were used in the mesh-independence investigation: coarse, normal, and fine. After the analysis, we discovered that the fine mesh produced precise findings and was of high quality. The mesh properties are listed in Table 4. Eight different grids were used to ensure that the results were independent of the grid. The indicators of the results are the total deformation and stress, as shown in Figs. 8 and 9. The fifth grid was chosen as the winning grid because of its varied results.

The fifth grid was selected because of these two variables: (1) maximum and minimum directional deformation (mm) and (2) maximum and minimum deformation (mm).

Conversely, the eighth grid was selected because of the following reasons: (1) improved and smoother convergence of solutions to appropriate criteria, (2) higher precision, (3) superior quality of structured multi zone mixed-cells meshes, and (4) shorter calculation time with realistic results. A professional ANSYS Workbench tool was used to assist in meshing the large machine tool. Under the circumstances of total deformation, element quality, and mesh of the six machine tools. The setup used in this study has the following specifications: Intel core i5 processor, 36 GB RAM, 45 min execution time, and 100 iterations.

## Analytical study

### Static structural analysis

**Figure 10:**
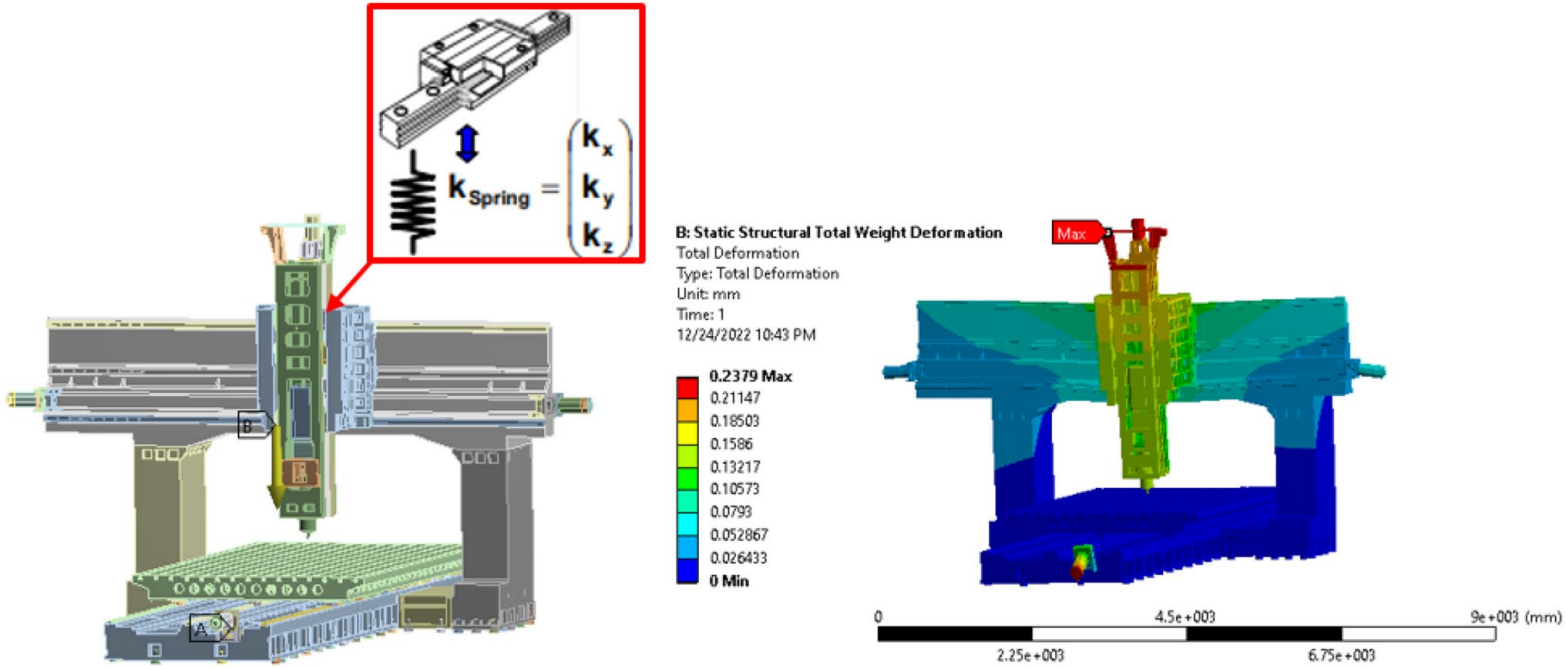
Total deformation according to static structural analysis.

Simulations of displacements, stresses, strains, and forces in structures or components caused by loads with negligible damping and inertia were performed using static analysis. Static analysis assumes that the loads acting on the structure and its reaction are stable; that is, they change gradually over time. Static analyses can be linear or non-linear. The linear static method was used when the structure underwent only a modest elastic deformation. It is assumed that the analysis refers to the standard method of computing elasticity problems, which can be represented analytically for extreme elementary structures. Significant deformations, flexibility (non-linear material qualities), creep, and yield stress are all examples of non-linear material properties, and contact (gap) elements and hyperplastic components are all permitted in non-linear analysis. Solid-body analysis is concerned with the system's equilibrium in global coordinates. Using ANSYS software, the finite element analysis approach provides answers for displacement, induced stresses, and strains caused by applied loads. The static analysis disregards damping and time-variant loads. The governing equations are as follows^48^:

1 $$\frac{\partial {\sigma }_{x}}{{\partial }_{x}}+\frac{\partial {\tau }_{xy}}{{\partial }_{y}}+\frac{\partial {\tau }_{xz}}{{\partial }_{z}}=0$$

2 $$\frac{\partial {\tau }_{xy}}{{\partial }_{x}}+\frac{\partial {\sigma }_{y}}{{\partial }_{y}}+\frac{\partial {\tau }_{yz}}{{\partial }_{z}}=0$$

3 $$\frac{\partial {\tau }_{xz}}{{\partial }_{x}}+\frac{\partial {\tau }_{yz}}{{\partial }_{y}}+\frac{\partial {\sigma }_{z}}{{\partial }_{z}}=0$$

where x, y, and z are the components of the normal stress, and xz and yz are the components of the shear stress. For the finite element approach, identical calculations were performed in matrix form.

4 $$\left[K\right]\left\{\delta \right\} -\left\{F\right\}$$

where [K] represents the element stiffness matrix, which contains the nodal displacement vectors of elements u, v, and w with respect to the global coordinates in the x-, y-, and z-axes, and F denotes the nodal force element vector in the x-, y-, and z-axes. The total displacement (deformation) owing to the applied load on the tool turret was 0.2379 mm, as shown in Fig. 10. The maximum and minimum deformations occur at the tool turret and base of the machine, respectively.

Meshed structural components are connected by spring elements with appropriate stiffness values, as opposed to guidance systems and drives, such as ball screws, which are commonly used to connect real structural components in machine tools. The stiffness of these springs is a good analog of the connections between actual machine parts^49^. They may additionally include local damping qualities if direct dynamic calculations are required and if they are known for the associated machine component. Boundary and load conditions were added to properly define the simulation model. Boundary conditions are used to describe the symmetry conditions and provide specified displacements. These are defined by securing various translational and rotational degrees of freedom or by the node of the restricted mesh. Using the findings of the static and harmonic analyses under a range of loading situations, the static and dynamic stiffness of the high-precision machine tool were measured on-site in each loading direction. This provides a clear basis for evaluating how well different materials function as structural components in massive machine tools. Structural deformations can affect the precision with which CNC machines, tools, and assembly lines place parts in the production process, particularly for larger systems. It may be challenging to model and accurately forecast the structural deformations that occur in the body of a machine. The static characteristics of the machine were obtained through static analysis and were used as a reference for subsequent structural improvements.

#### Static analysis with a 5000 N X-axial cutting force

The stiffness of a machine tool is an important index to consider. The machining accuracy is negatively affected if it does not have sufficient stiffness. When heavy cutting is performed, the effect is significant if the stiffness is poor. The boundary condition for the analysis was to apply a force of 5000 N in the x-axis to a test tool holder located 100 mm below the spindle nose.

**Figure 11:**
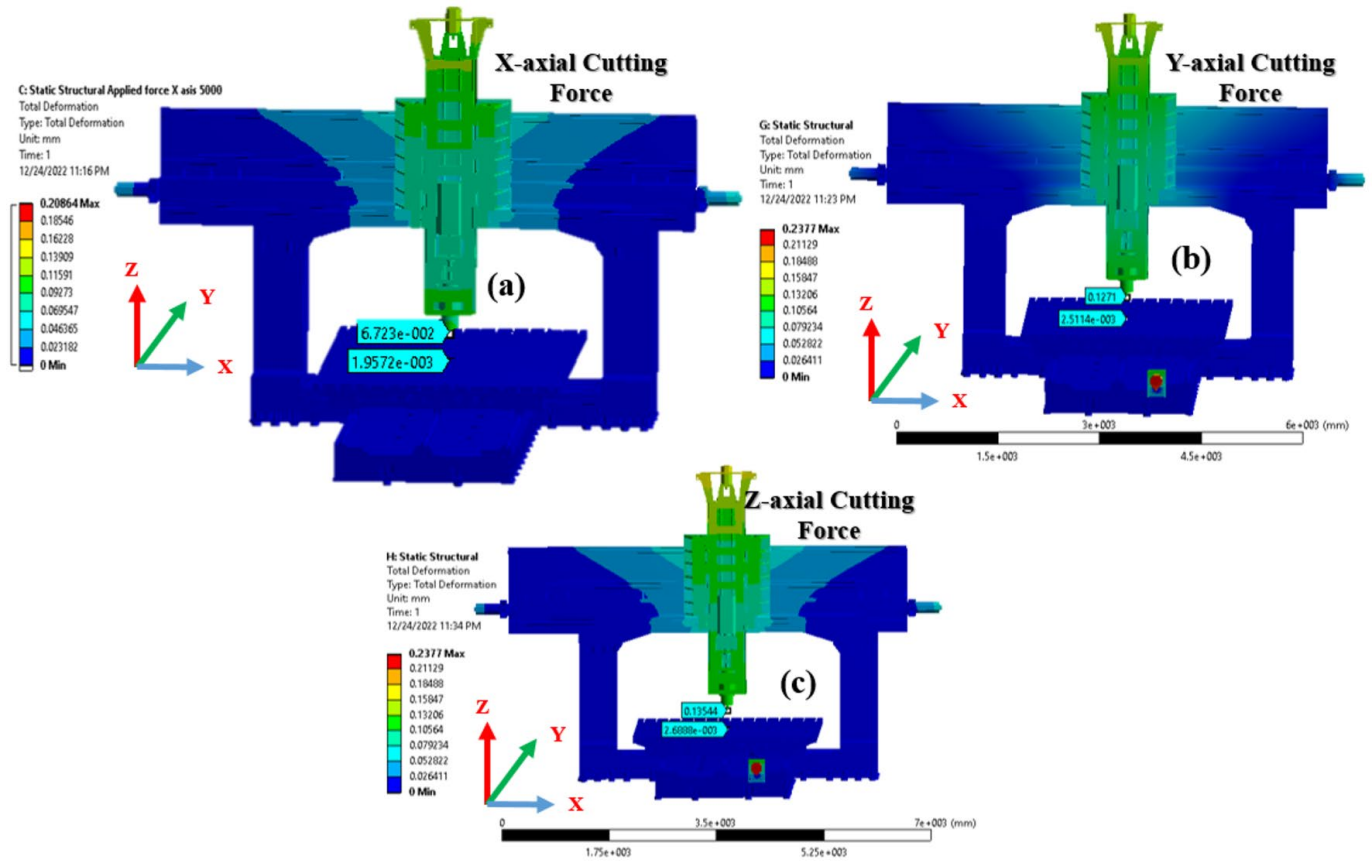
Static structural analysis with a (**a**) 5000 N x-axial cutting force (**b**) 5000 N y-axial cutting force (**c**) 5000 N z-axial cutting force.

The findings of the analysis are presented in Fig. 11. The amount of deformation experienced by the spindle end was 67.26 μm, and that of the bed was 1.95 μm. Therefore, the spindle head is inferred to be susceptible to deformation when an external force is applied; this is because the spindle head undergoes a degree of deformation that is larger than that of the bed, making necessary alterations to the structure.

#### Static analysis with a 5000 N Y-axial cutting force

The analysis boundary condition was to apply a 5000 N force in the y-axis to a test tool holder situated 100 mm below the spindle nose. The results of this analysis are depicted in Fig. 11b. The amount of deformation experienced by the spindle end was 127 μm, whereas that of the bed was 2.5 μm.

#### Static analysis with a 5000 N Z-axial cutting force

The machine was studied in a static state, and the applied load on the spindle nose was maintained at 5000 N in the z-axis, comparable to that in the preceding study. The only criterion that differed was the fixed-support region. The boundary conditions, such as gravity and the amount of external force, were the same. The results of the analysis are shown in Fig. 11c. Deformations of 135 μm and 2.68 μm were observed at the spindle end and bed, respectively.

### Experimental validation of static analysis

**Figure 12:**
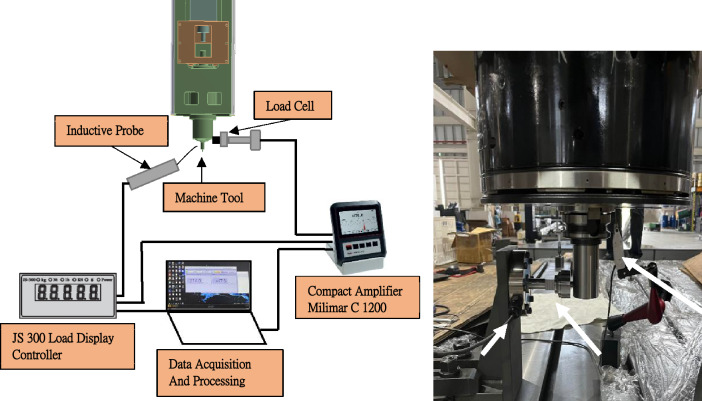
Experimental apparatus.

Stiffness is an essential characteristic of a manufacturing equipment because it contributes to its ability to accurately maintain the position between a tool center point and work-piece under stress^50^. In a previous case study, the stiffness response of the machine tool was discovered to vary by up to 27%^51^. To enhance the static stiffness of the machine, the type of ground support upon which it rests was modified. The shift in the frequency spectrum was determined by performing modal analysis on each model. To assess the stiffness of the machine, a structural analysis was performed. This experiment primarily focused on testing the x-axis stiffness of the entire machine. The test technique consists of the spindle clamp of a test tool holder and the force-applying fixture being linked to the load cell and secured to the bed. The force was applied 100 mm below the spindle nose. The position of the meter was above the nose of the spindle. To ensure the reproducibility of the experiment, an external force of 150 kg was applied to the spindle thrice during the experiment. The experimental apparatus is shown in Fig. 12.

#### Leverage scale

Inductive probes are intended for machine-tool use. The following measures were used: path, distance, thickness, and machine component position detection. The sensors (probes) were used only within the value range defined by the technical data. The leverage scale specifications are listed in Table 6.

**Table 6:** Leverage scale specification.

Measuring range (mm)	± 0.3
Measuring force (N)	0.25 ± 0.5
Distance of lower stop MAX	− 0.37
Distance of upper stop MAX	1.6
Sensitivity deviation	0.5
Repeatability fw (µm)	0.03
Measuring value hysteresis fu (µm)	0.5
Linear deviation within +/− 0.3 mm	0.9
IP protection category	IP 50

#### Load source

The most common force measurement load cell is the LM load cell. Load induction is maximized owing to the large area, stable base, and extremely low height, combined with the high-efficiency shear induction structure and complete sealing protection.

### X-Axis rigidity test of the entire machine

#### Result verification

**Figure 13:**
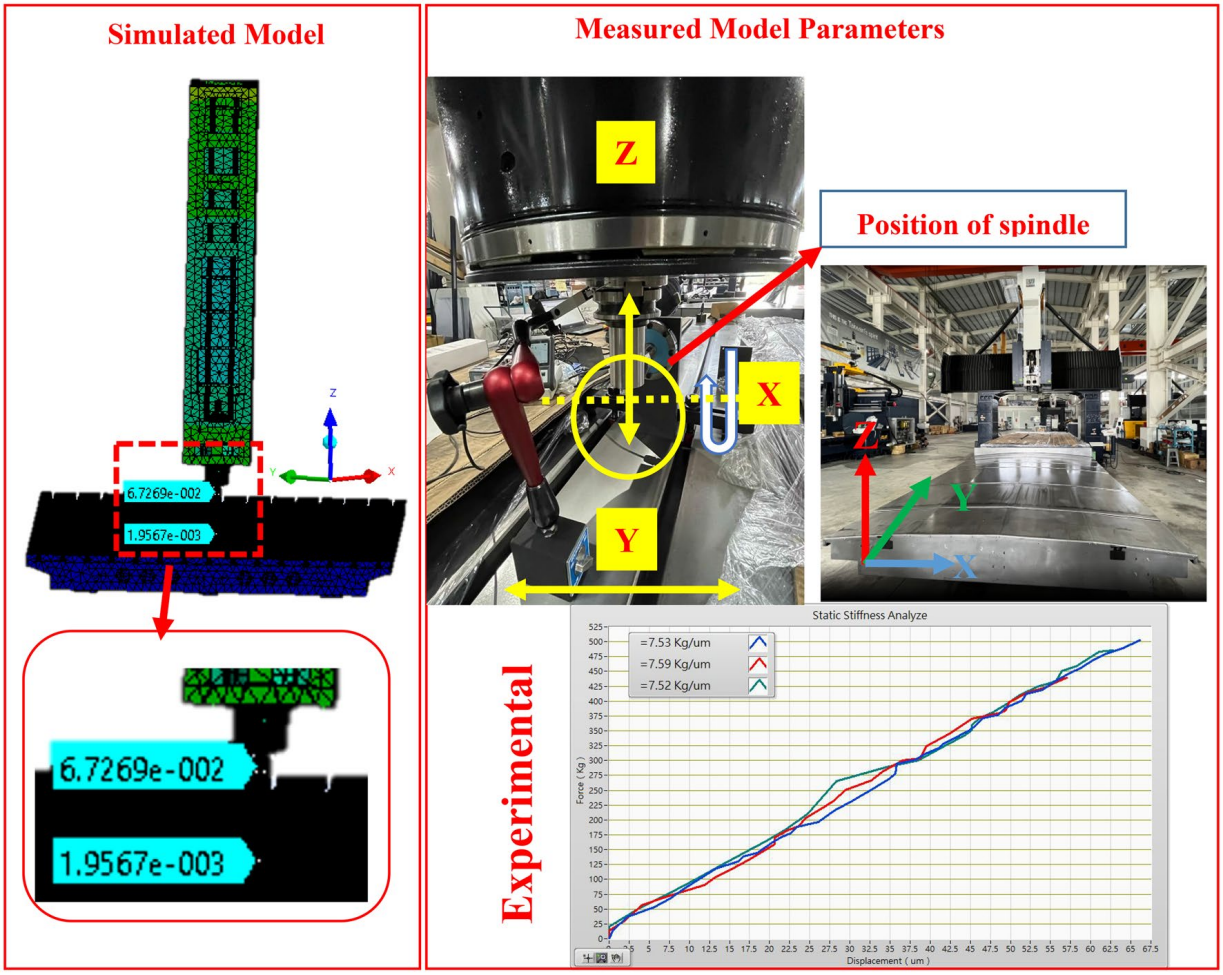
X-axis rigidity test of the entire machine.

During testing, the force was applied by means of the load source, whose specifications are listed in Table 7. Data on the resulting displacement were recorded using a data-acquisition equipment. A leverage scale was used to calculate the amount of deformation. A gradual increase in force was used to monitor the effects of the deformation and forces. The process was repeated thrice to ensure consistency. Figure 12 illustrates the results of the studies performed at the applied load position with the experimental setup. Figure 13 depicts the assembly of the x-axis stiffness measurements performed on the machine. The primary components include a gauge, load cell, and mechanism for the force application. Figure 13 shows the results of the measurements taken in the graph of the experimental values. It is clear that the outcomes of the three separate trials were comparable to one another. From 0 to 500 kg, there is a linear increase in the amount of deformation. After averaging the results of three separate measurements, the x-axis stiffness of the entire machine was determined to be 7.53 kg/μm.

**Table 7:** Specifications of JS-300 load display controller.

Load cell loading voltage (V)	DC 5
Input range (mV/V)	0.5–4
Operating temperature (°C)	0–50
Serial output	RS-232

According to the static analysis results, a force of 5000 N was applied in the x-axis of the spindle head. The deformation of the spindle nose was 67.26 μm, and the deformation of the bed was 1.956 μm. The relative deformation of the table is 7.53 μm, and the rigidity of the entire machine in the x-axis is 7.65 kg/μm. According to the static rigidity experiment, the rigidity of the entire machine in the x-axis is 7.53 kg/μm, as shown in Fig. 13. Therefore, the error between the static analysis and static rigidity experiment is 1.56%. The RCFM was used to design the structure of a precision machine tool by considering how the rail and cylinder of the linear guide touched each other. Subsequently, it was used to solve the related problem of structural optimization. First, a CAD model was used to create the FE model, which had a mesh of approximately 1,499,510 elements. The structural parts of the assembly were made of structural steel and cast iron, the properties of which are listed in Tables 2 and 3. The contact elements in the FE library were used to create other connections outside the design domain. The FE model was attached to the ground, and structural loads were placed on the tip of the spindle. Above Fig. 6b shows a simplified version of the three-axis machine tool used to construct the model.

## Model analysis

The ANSYS Workbench software was used to model and analyze the finite elements at the same machining position to obtain the simulated modal parameters. Modal analysis is mostly used to examine the natural frequency of the entire machine. The machine, as well as its vibrating and weak dynamically stiff components, may produce significant vibrations when it operates at its inherent frequency. The weak component of the precision machine tool structure is a type of energy concentration that can indicate a change in the dynamic character of the machining process^52^. During the machining process, the degrees of freedom with wide amplitudes of modal mass distribution are referred to as weak machine components^53^. For the free vibration of the machine tool structures, we have the following dynamic equation:

5 $$M\ddot{x}+C\dot{x}+Kx=0$$

In this case, the equivalent characteristic equation is as follows:

6 $$[{\lambda }^{2}M\ddot{x}+\lambda C+K]\varphi =0$$

The modal mass of each mode can be calculated using the eigenvector; for example, the modal mass of the i-th mode is given by^49^

7 $${m}_{i}={\varphi }_{i}^{T}M{\varphi }_{i}|=[{\varphi }_{1i }{\varphi }_{2i }\cdots { \varphi }_{ni}]\left[\begin{array}{ccc}{M}_{1}& \cdots & 0\\ \vdots & \ddots & \vdots \\ 0& \cdots & {M}_{n}\end{array}\right]\left[\begin{array}{c}{\varphi }_{1i}\\ {\varphi }_{2i}\\ \vdots \\ {\varphi }_{ni}\end{array}\right]=\stackrel{n}{\sum_{n=1}}{M}_{k}{\varphi }_{ki}^{2}$$

where n is the number of degrees of freedom in the structure, $k$ is the $k$-th degree of freedom, and ${M}_{k}{\varphi }_{ki}^{2}$ represents the distribution of kinetic energy in the $i$-th mode across the $k$-th degree of freedom. The modal mass distribution for each degree of freedom in the $i$-th mode can be described as follows:

8 $${m}_{i}^{\mathrm{^{\prime}}}=[{M}_{1}{\varphi }_{1i}^{2}{M}_{2}{\varphi }_{2i}^{2}\cdots {M}_{n}{\varphi }_{ni}^{2}{]}^{T}$$

9 $${M}^{\mathrm{^{\prime}}}=[{m}_{1}^{\mathrm{^{\prime}}}{m}^{\mathrm{^{\prime}}}2\cdots {m}_{n}^{\mathrm{^{\prime}}}]$$

Only the first few natural frequencies and associated vibration types are important for this system. The dynamic structure of the machine tool was investigated using the FE method at frequencies between 0 and 1000 Hz. The natural frequency of the machine, according to the findings of the modal analysis, are 22.5, 28.9, 40.0 and 47.0 Hz, respectively.

**Figure 14:**
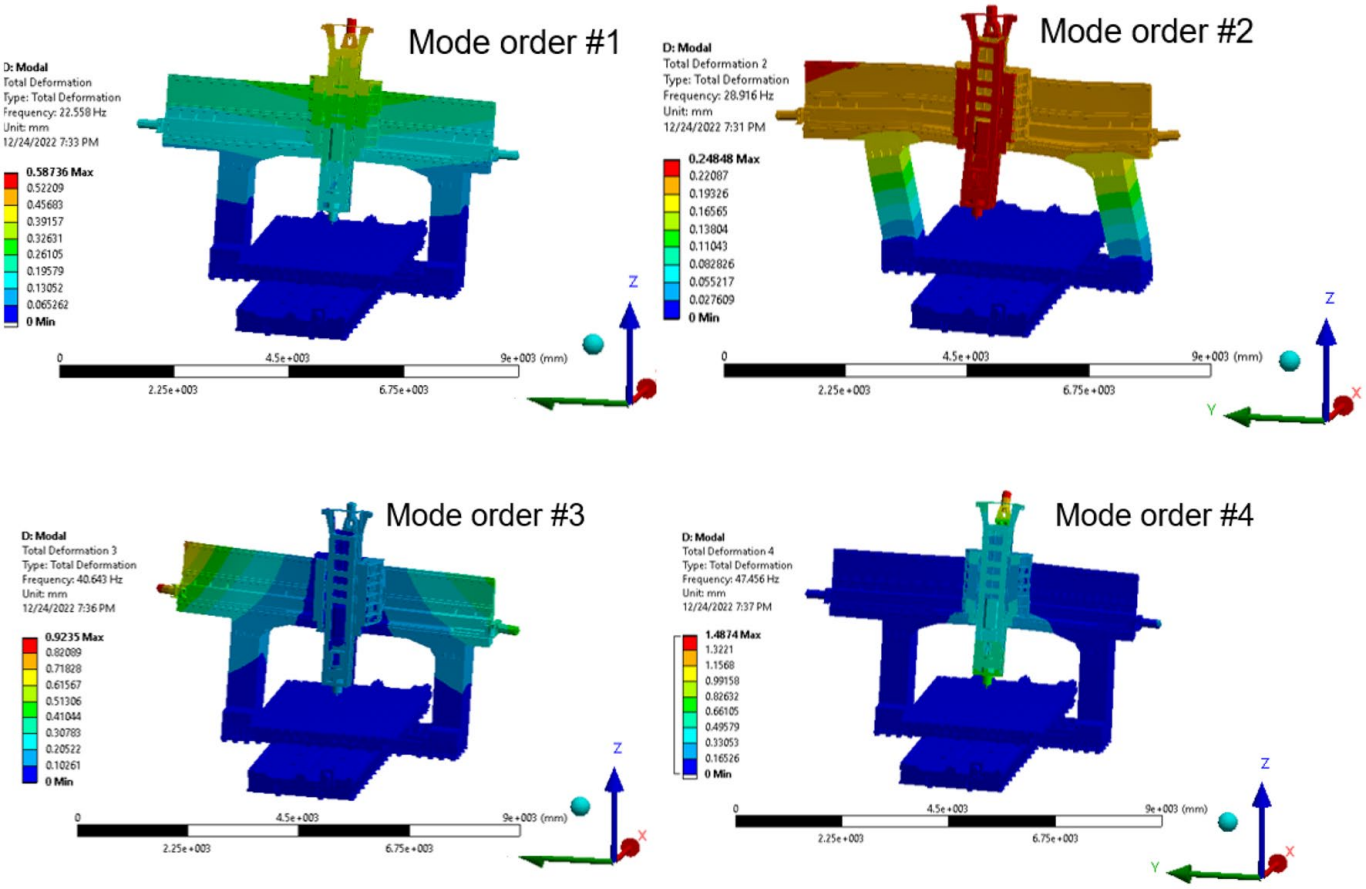
Mode shapes of the machine tool structure.

Modal analysis was used to identify the shapes and frequencies of the fundamental vibration modes. In this study, the first four natural frequencies and their vibration modes were extracted. Figure 14 shows the natural frequency of the precision machine tool structure under different modes. In the 1st mode shape, the gantry swung back and forth. In the 2nd mode, the gantry swung from side to side. In the 3rd mode, the frequency mode shape is the gantry torsion left and right. Finally, in the 4th mode shape, the gantry does not exhibit any significant change in shape. Subsequently, the finite element model was used for modal analysis to satisfy the accuracy requirements. Hong et al.^54^ performed a static structural analysis on a turning milling complex CNC machine to validate the originality of the design and recommended an optimal method to minimize the machine's weight to further optimize its machining process. This study used FEA to analyze the design attributes in a similar manner. The effect of steady-state loading circumstances under the influence of standard gravity without a time constraint was evaluated in static structural analysis, which was performed on the machine to determine the part displacement and machine static rigidity. The behavior of the machine was evaluated when an external force of 5000 N was applied to the tool. A transient structural analysis was used to assess the response of the machine to time-dependent loads. The harmonic response of the tool turret was examined to analyze the vibrational excitations under the applied load, as machining involves the effect of force on the tool.

## Harmonic analysis

**Figure 15:**
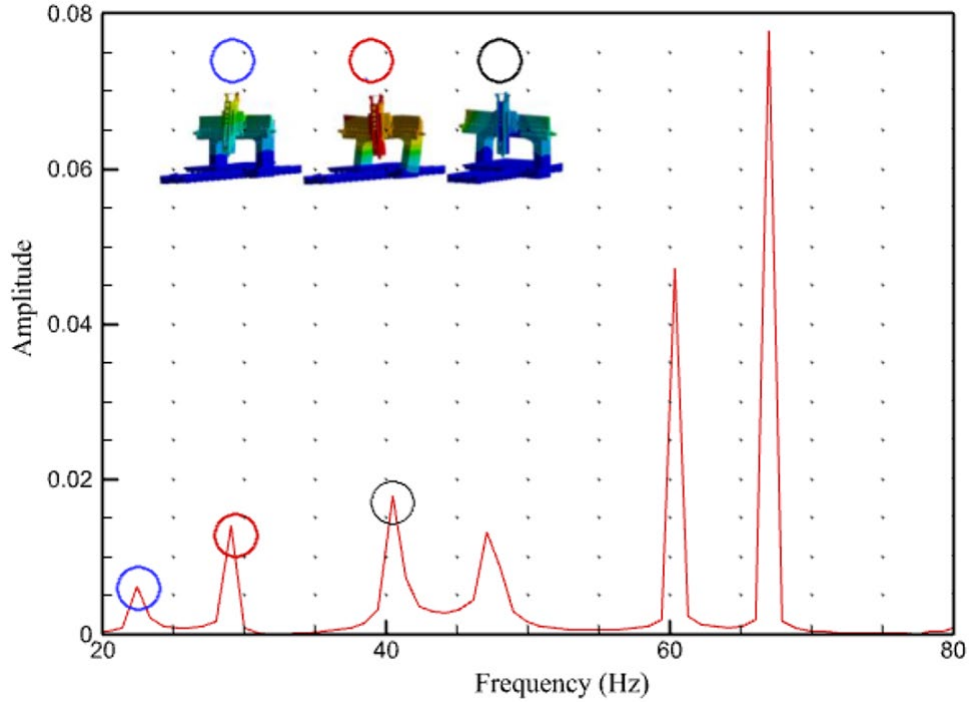
Harmonic response.

We can analyze the response of the machine in each axis once an external force is applied using spectrum analysis. In this case, an external force of 5000 N was applied to the spindle nose. Hong et al.^54^ performed a static structural analysis on a turning milling complex CNC machine to validate the originality of the design, and provided an effective way to minimize the machine's weight to further optimize its machining process. This study used FEA to analyze the design attributes in a similar manner. The effect of steady-state loading circumstances under the influence of standard gravity without a time constraint was evaluated in static structural analysis, which was performed on the machine to determine the part displacement and machine static rigidity. The behavior of the machine is evaluated when an external force of 5000 N was applied to the tool in the x-axis. A transient structural analysis was used to assess the response of the machine to time-dependent loads. The harmonic response of the tool turret was examined to analyze the vibrational excitations under the applied load, as machining involves the effect of force on the tool. According to the modal testing technique, the machine was excited and its reaction was recorded using the ANSYS Workbench. Excitation was accomplished by applying force. The impact point of the force was comparable to the experimental testing point given that the point adequately stimulated the machine. The component is identical to that employed in the modal analysis because the excitation range is utilized to determine the findings of the modal analysis. Based on the results of the modal analysis, the operating frequency range was 100–200 Hz. The frequency response of the tool turret was then determined. A total of 250 solution intervals were plotted to represent one hertz of frequency. As shown in Fig. 15, the machine responds at a specific frequency, as indicated by a change in the phase angle**.**

The excitation of the machine at a specific frequency is indicated by clear peaks in the regions of 22, 28, and 38 Hz. These results are comparable to the frequency results generated in the modal analysis. The findings substantiate both the validity of the modal analysis and the precision of the results obtained.

## Topology optimization

**Figure 16:**
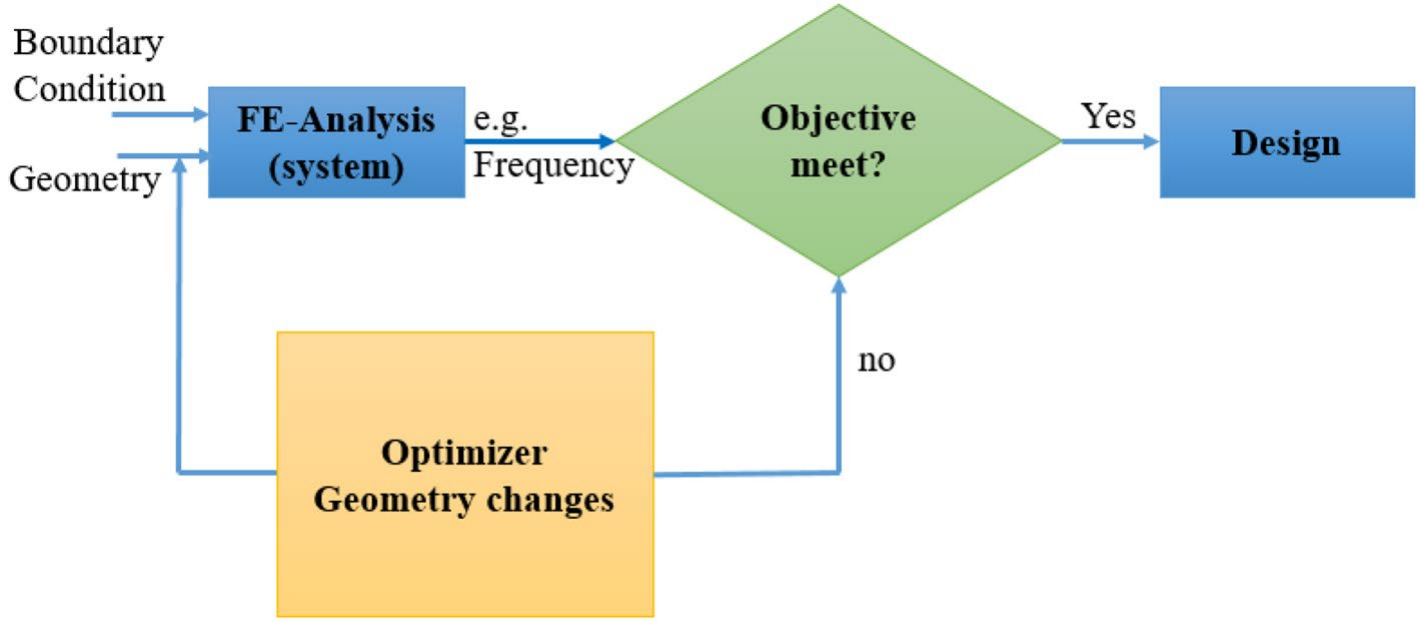
Optimization of the controller component.

**Figure 17:**
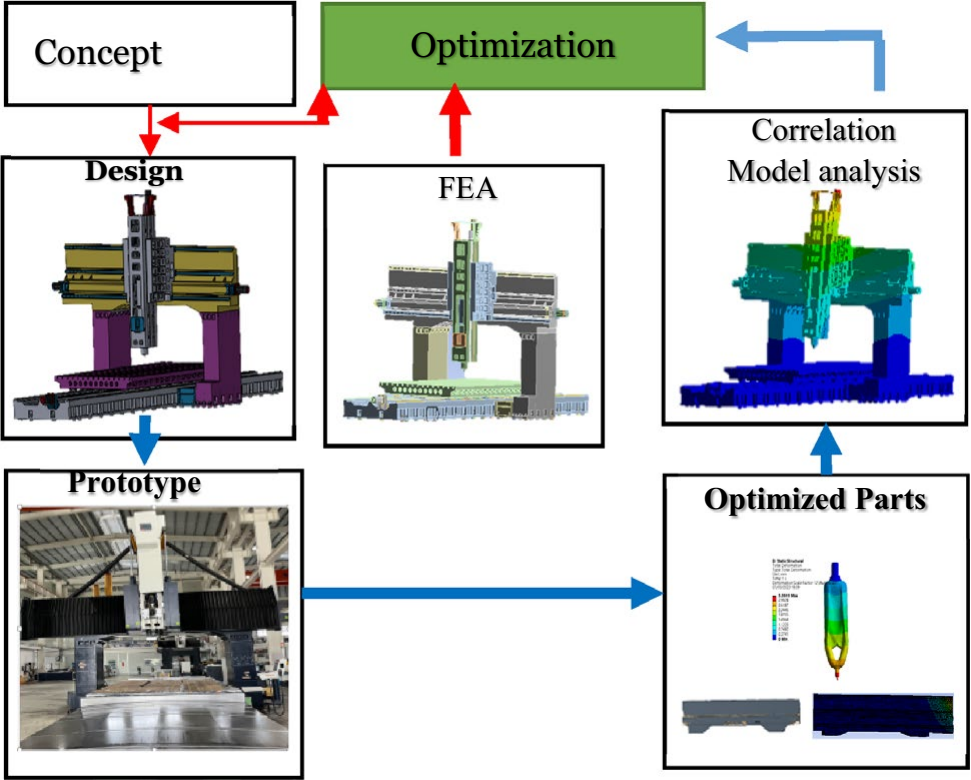
Simulation and optimization integrated in the design process.

Owing to the abovementioned challenges, studies on contact-coupled issues are uncommon in the literature. The SIMP method is used in the majority of these studies, which involves formulating the issue mathematically by combining optimization with HSM frictionless contact conditions^55–57^. Using the ANSYS optimization technique, four objectives were created for this procedure. The four natural frequencies of the gantry were first estimated and related to the efficiency of the machine parts and mass of the gantry. The inherent frequencies of the machine must be increased while the gantry mass must be reduced, as shown in the flowchart in Fig. 16. The performance of a machine is correlated with its natural frequency if its mass is typically opposite to that of the structure's high natural frequency under post-optimization conditions. Utilizing modern optimization techniques based on the finite element method makes it possible to accomplish these gains in machine performance at a reasonable cost, as shown in Fig. 17. The following section describes and discusses these strategies.

For the optimization process, a few gantry dimension parameters were determined. These factors were altered throughout the process to produce a new design with new proportions. Additionally, the dynamic and static behavior findings of the new design were examined using ANSYS, and the objectives were optimized. The strategic gantry machine tool market is highly competitive, with many manufacturers devoting resources to the research and development of high-speed high-precision gantry machine tools. This has necessitated the development of several new features during the gantry machine tool design stage.

In this study, the machine tool's workbench model and cross rail were optimized, and the reworked model was imported into the finite element analysis software for static and modal analyses to compare the differences. Subsequently, the modified model was combined for the entire machine analysis, and the results were compared.

### Optimality criteria method

In the discrete topology optimization problem, there are many design variables, which are denoted as D herein. Therefore, it is common to use iterative optimization techniques, such as the optimality criteria method, to solve this problem^58^. Gilles Foucault^59^ suggests an innovative topological data-structure for FE model preparation that incorporates interior geometry adjacency links in MC faces, a set of topology editing operators, and a set of adaptation criteria. First-Order Bilevel Topology Optimization (FBTO), as proposed by Zherong Pan^60^ can tackle a variety of important issues in the robot design paradigm, such as Topology Optimization under self-weight and numerous external loads. For a series of 2D and 3D problems, we analyze and contrast FBTO with earlier Topology Optimization solvers. In order to evaluate this CAD model, Abdennour Amroune^61^ performed the Finite Element Analysis (FEA) on both the 3D optimum shape and the CAD solid model derived from that as well. Amir M. Mirzendehdel^62^ identified significant complex designs that can be 3D printed and post-processed for support removal using a specialized combination of tools and fixtures. Several case studies are presented to illustrate the efficacy and utility of this new method. Using of technique works on nontrivial 2D and 3D instances. Using the optimality criteria method, the design variables are updated at each iteration using a heuristic scheme. The Lagrange for the optimization problem is:

10 $$L({\mathrm{x}}_{\mathrm{i}})={\mathrm{u}}^{\mathrm{T}}\mathrm{Ku}+\Lambda ({\Sigma }_{\mathrm{i}=1}^{\mathrm{n}}{x}_{i}{\upsilon }_{i}-{\mathrm{V}}_{\mathrm{o}})+{\uplambda }_{1}(\mathrm{Ku}-\mathrm{F})+{\Sigma }_{\mathrm{i}=1}^{\mathrm{n}}{\lambda }_{2}^{i}({\mathrm{x}}_{\mathrm{min}}-{\mathrm{x}}_{\mathrm{i}})+{\Sigma }_{\mathrm{i}=1}^{\mathrm{n}}{\lambda }_{3}^{i}({\mathrm{x}}_{\mathrm{i}}-1)$$

where ${\uplambda }_{1}, {\uplambda }_{2},\mathrm{ and }{\uplambda }_{3}$ are Lagrange multipliers for various constraints. The ideal situation is given by

11 $$\frac{\partial \mathrm{L}}{\partial \partial {\mathrm{x}}_{\mathrm{i}}}=0,\mathrm{i}=\mathrm{1,2},3,\cdots ,\mathrm{n}$$

which leads to the observance of the Rule of Law:

12 $$\mathrm{L}={\mathrm{u}}^{\mathrm{T}}\mathrm{Ku}$$

The optimality condition can be written as follows by differentiating Eq. (12)

13 $${\mathrm{B}}_{\mathrm{i}}=\frac{\frac{-\partial \mathrm{C}}{\partial {\mathrm{x}}_{\mathrm{i}}}}{\Lambda {\upnu }_{\mathrm{i}}}=1$$

The following equation can be used to calculate conformity sensitivity:

14 $$\frac{\partial \mathrm{C}}{\partial {\mathrm{x}}_{\mathrm{i}}}=-\mathrm{p}({\mathrm{x}}_{\mathrm{i}}{)}^{p-1}{u}_{i}^{T}{K}_{i}{U}_{i}$$

The design variables will then be updated based on the following equations.

$${\mathrm{new}}_{\mathrm{xi}}=\left\{\mathrm{max}\left({x}_{min}-m\right),{\mathrm{ifx}}_{\mathrm{i}}{B}_{i}^{\eta }\le {\mathrm{x}}_{\mathrm{min}},\left({\mathrm{x}}_{\mathrm{min}}-\mathrm{m}\right)\right\}$$

15 $$=\left\{{\mathrm{x}}_{\mathrm{i}}{B}_{i}^{\eta },\mathrm{if max}\left({\mathrm{x}}_{\mathrm{min}}-\mathrm{m}\right)<\mathrm{min}\left(1,{x}_{i}+m\right)\right\}$$

16 $$=\left\{\mathrm{min}\left(1,{x}_{i}+m\right),\mathrm{ifmin}\left(1,{\mathrm{x}}_{\mathrm{i}}+\mathrm{m}\right)\le {\mathrm{x}}_{\mathrm{i}}{B}_{i}^{\eta }\right\}$$

In design process, utilizing machine tools, also known as deformation tools, involves high material costs while also requiring conditions of high resistance to deformation. The reduction of the mass of the parts, i.e. the reduction of the costs with the materials, from the composition of the tools, while maintaining constant maintenance, or, if possible, by increasing the rigidity, which is identical with the decrease of the metallic materials. When attempting to optimize the shape of mechanical parts. The load was applied to the top of the part to simulate the “action” that causes permanent deformation. The Boundary conditions, or fixed supports, are materialized in the piece's lateral face and upper edge of the opposite face. Both the applied load and the limit conditions simulate real-world industrial situations. The primary purpose of topology optimization is to solve the PDE issue and identify the optimal solution for rational and efficient Tool, Rail, and column material consumption in the given loading condition. In order to minimize the mass, areas that contribute less to functionality and maintain higher stress values are eliminated from the initial geometry by modifying the geometry of the component in accordance with the distribution of equivalent stress.

### Identifying the shape optimization technique

If we assume a finite, well-defined domain Ω ⊂ ${\mathrm{R}}^{\mathrm{d}}$.To resolve the PDE issue, or to offer the mathematical model's best solution, way to locate a subspace ⊂ Ω minimizes the objective function J, which functions as a function of ω^61^.

17 $$\mathrm{min}J(\omega )$$

18 $$\omega \epsilon {U}_{\alpha d}$$

where ${U}_{\alpha d}$ represents the subdomain reunion of legal forms amongst subdomains Ω. Optimization technique may include user-defined limitations. The purpose of function, J, as function of ω for find the PDE problem. Defining the cost function, J, and the range of allowable values for the subdomains (within the constraints) is fundamental. Numerical scholars^62,63^ suggest unified goal functions (due to their valuable properties rather than their fidelity for solving the PDE problem or, in general, for the applications), whereas real-world applications expect complicated and not-so-common approaches, so the objective functions are neither simple nor approachable. Some examples of such limitations include: maximum acceptable local stress, normal force, operational life, minimum curve radius, refrigeration of the injection component in less or more time than specified, and so forth. Several of these issues are even difficult to solve or affordable mathematically in a common method (for example, limitations connected to the casting process) and can make it impossible to solve or need the solution of additional complex problems.

### Objective function and methodology

“The compliance of the elastic element is defined by the inverse of the stiffness.” George I. N. Rozvany et al.^61^ optimized many objectives with the cost function belonging to the stress

19 $$J\left(\omega \right)={\int }_{\Omega }{\left|\sigma \right|}^{p}$$

Find the optimum compromises for a structure continuously loaded.

20 $$J\left(\omega \right)=\sum_{i }{C}_{i}{\int }_{\Omega }X\left(x\right)Ae\left({u}_{i}\right)dx$$

21 $$= {\sum }_{i}{C}_{i}{\int }_{\Omega }{A}^{-1}{\sigma }_{i}:{\sigma }_{i}dx$$

22 $$= {\sum }_{i}{C}_{i}{\int }_{\partial\Omega }{f}_{i}. {u}_{i}ds$$

Or the Von Mises stress equivalent. In this work, we used the Von Mises stress distribution, which can be studied using the ANSYS-Mechanical model. In this paper, we focused on designing and developing an integrated analysis for optimizing the topology of the machine spindle tool and rail part in the composition of a plastic deformation mold, as well as validating the geometry acquired after optimization. The complete structural analysis-topological optimization-geometry validation-parametric optimization approach was carried out using ANSYS FEA packages and the design of experiments (DOE) module. The part's behavior in a static structural system was examined (the outcomes were total deformation and equivalent stress von misses) as well as the part's life, or safety factor. With the necessary settings for re-configuring the part, an approximate, technological sketch was obtained in the Space Claim CAD system using specialized software, a component of ANSYS, the dimensions of which were modified by the Structural Optimization system to respect the loading conditions and border conditions. The geometry obtained after optimization served as the starting point in the structural analysis for validating the pre-optimization system's state variables, establishing and defining the output variables (parameters) (in Space Claim-output variables (parameters), being linear dimensions), respectively in Mechanical Model (output parameters: part mass and Total deformation). To respond to the inquiry, “What is the best design point for getting the best project?” we use these parameters (input and output parameters) to locate the design points space, generate a Response Surfaces, and optimize the project based on Candidate Points (within the bounds of having the lowest possible mass and the lowest possible compliant and total deformation, before the optimization is achieved). The completed model that emerges from the iterative optimization process must be operational under the given conditions and limitations. Topological optimization and topographic optimization are two of the extant approaches of structural optimization that have been applied thus far. Topological optimization allows for the volume of the material to be reduced while the stiffness of the improved model is increased. The main parts machine tool and cross rail shown in Table 8.

**Table 8:** Optimized machine parts specifications.

Optimized parts		
Part name	Material	Weight
Machine tool	Structural steel	138.9 kg
Cross rail	Gray cast iron FC300	11,466 kg

To confirm that the created product satisfied the requirements, the newly designed model was assessed using computer-aided analysis, and the model was optimized using structural optimization. The model optimization deleted extraneous pieces, lowered production costs, and enhanced the model's overall static and dynamic rigidity. The following are the findings of this study based on finite element machine topology optimization.

**Figure 18:**
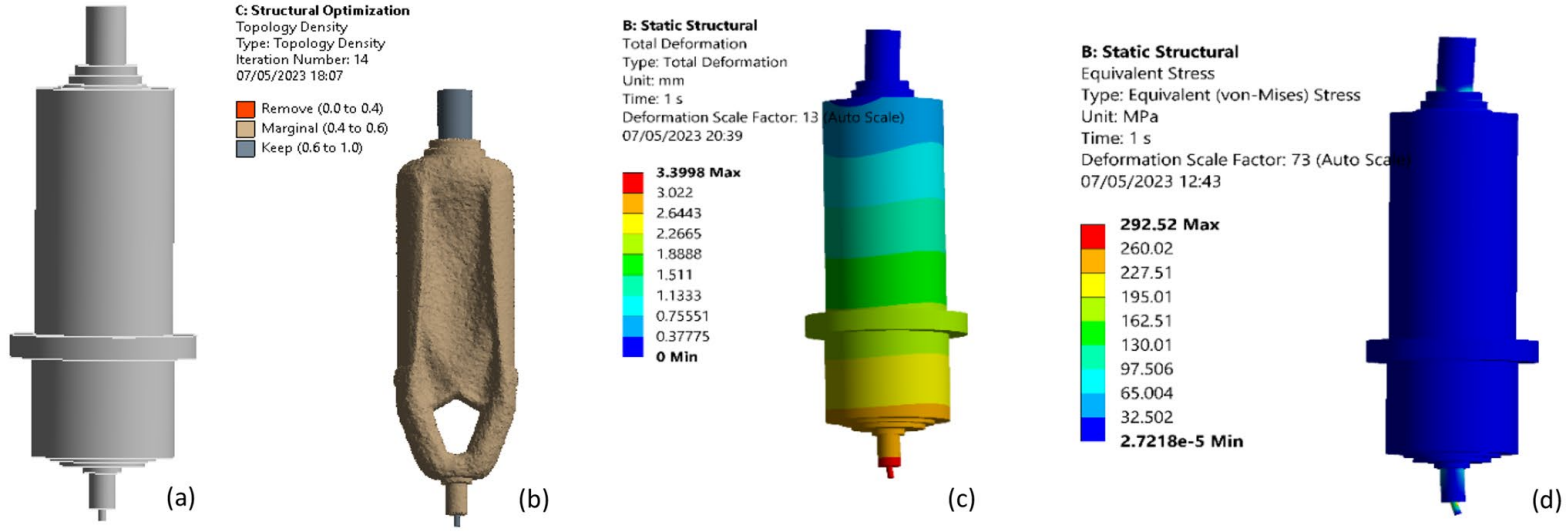
(**a**) Machine spindle tool, (**b**) spindle tool representation of the optimized part, (**c**) Initial model total deformation, and (**d**) Initial model von misses equivalent stress.

With finite elements, the optimized range (surface, volume of a part) is broken up, and then the conditions and applied load on tool tip 5000 N are put on the limit. The case study tool presented weight 138.91 kg and is composed of Structural Steel which is shown in Fig. 18a. Figure 18b shows an aspect of the material retention process beginning in the region of resistance to deformation (von Mises equivalent 292.52 stress and total deformation 3.399 mm) and resulting in the optimal shape with the percentage of retaining 85% in the initial volume, which was determined using topological optimization which is shown in Fig. 18c,d respectively.

**Figure 19:**
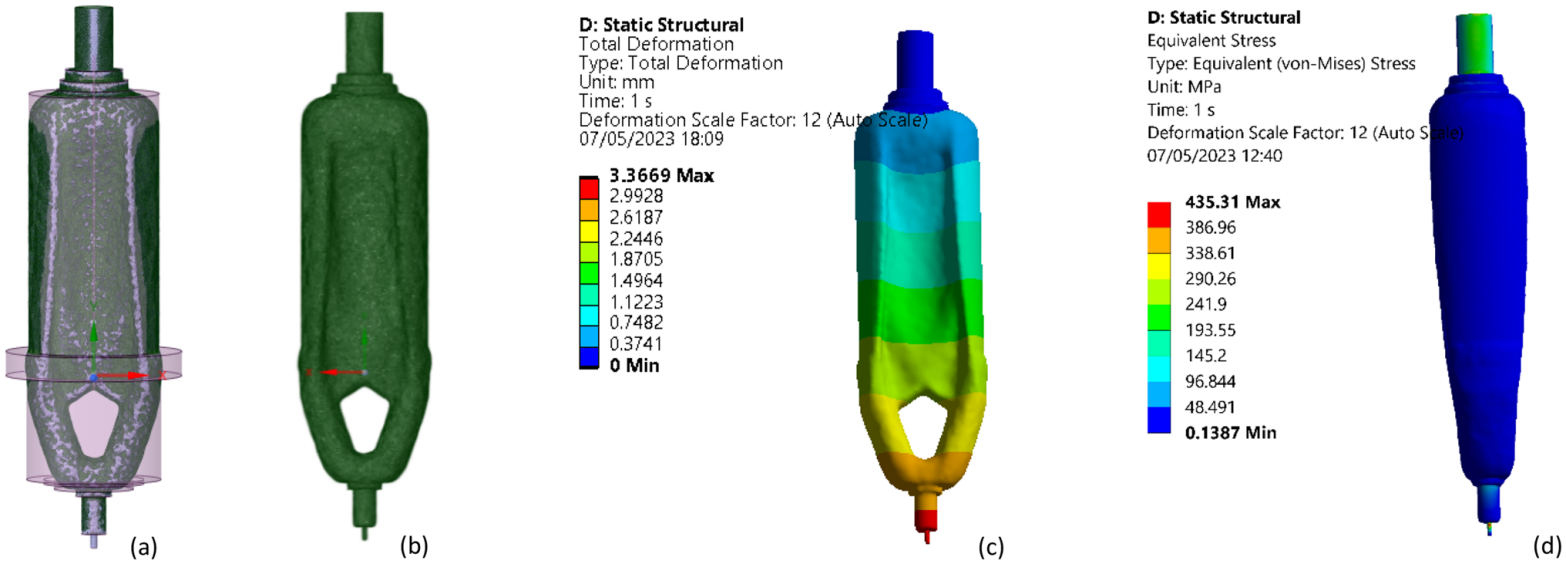
(**a**) Space claim-output model, (**b**) optimized model, (**c**) optimized model total deformation, and (**d**) optimized model von mises equivalent stress.

**Figure 20:**
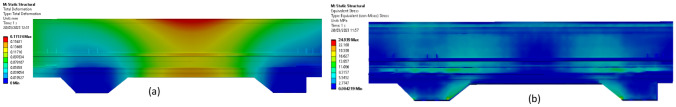
(**a**) Initial model total deformation of cross rail and (**b**) Initial Optimized model von misses equivalent stress of cross rail.

With the relevant settings for re-configuring the paoptimizationace Space Claim CAD system which is shown in the Fig. 19a,b, specialized software from ANSYS, an approximate technological sketch was created. After reducing the 70.68 kg from the machine tool after optimization upper solution value by total deformation is 3.366 mm and von Mises equivalent 292.52 stress which showing in Fig. 19c,d respectively, maximum value of comparable stress is 435.31 Mpa. The continuous optimized model must match product operation requirements. Topological and topographic optimization is utilized for structure optimization. Topological optimization reduces material volume and increases model stiffness (Fig. 20).

**Figure 21:**
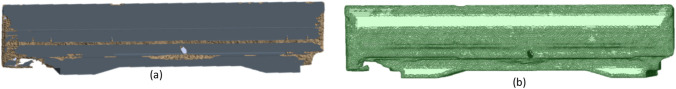
(**a**) Optimized model cross rail and (**b**) space claim-output model of cross rail.

Another method for enhancing the rigidity of the cross rail is to run a topology optimization analysis in ANSYS. Modal frequencies and stress–strain contours are studied on the cross rail. Topology optimization minimizes machine weight while maintaining design attributes. To avoid modifying too much of the design, the analysis is designed to restrict 85% of the actual mass as a precaution. The analysis shows Fig. 21 (a) Optimized Model (b) Space Claim-output model the area that is not stressed by the weight of the parts lying on it. To clear the highlighted region, an engineering design can be employed; the updated design is reanalyzed, and the results are compared. It determines the reduced mass and deformation under a specified shape. Now, the revised shape, as validated by the FEA package, has some material eliminated from areas that would not only sustain high stress but also bear load. It was effective to remove this surplus mass because we can still acquire a shape optimized for less stress and deformation.

**Figure 22:**
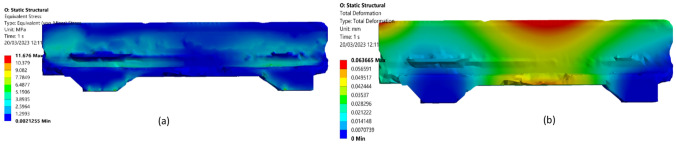
(**a**) Optimized model total deformation of cross rail and (**b**) optimized model von misses equivalent stress cross rail.

Boundary conditions, load direction, or load systems affect material distribution. After reducing the weight is 9363.2 kg from the cross rail static structural study showed that the maximum overall deformation is 0.17574 mm shown in Fig. 20a. Maximum von Mises equivalent stress is 24 MPa shown in Fig. 20b. The goal is typically to minimize the maximum equivalent stress in a structure. This means that the equivalent stress should decrease as the optimization process progresses, as the structure is refined and modified to better meet the design criteria. Total deformation is 0.063366 mn and von Mises equivalent stress is 11MPA after optimization which shown in Fig. 22a,b. Topology optimization was used to increase the quality of the machine, which in turn improved the quality of the surfaces of the parts that were machined. This was accomplished by lowering production costs and improving the quality of the machine itself.

## Conclusion

Among the several analysis and simulation methods, the key components are modeling, system modeling, static analysis, and dynamic analysis. FEA is a technique that is frequently used for both analysis and simulation. We can attain the following inferences by performing finite element modeling, static analysis, model analysis, and comparison with experimental static rigidity analysis using ANSYS Workbench. Based on model analysis, we discovered the first three distinct frequencies, 22, 28.5, and 40.1 Hz, as well as the type of vibration that indicates a weak link. An additional static structural investigation revealed that the degree of deformation of the spindle is 160 µm. We then performed an experimental investigation of static stiffness and obtained the experimental deformation values for both the tool and spindle which were 6.5 kg/μm. Based on the transient analysis results, the tool nose transient reaction was 0.5 s when a force of 5000 N was applied to the spindle nose. Comparing this with the FEA results verified the data quality of both the static and dynamic properties as well as the accuracy and effectiveness of the constructed finite element model. The difference in error was no more than 1.2% between the static ANSYS and experimental values. The fundamental objective of this study is to compile a list of capabilities of large gantry-type precision machine tools, such as their structural composition, static and dynamic features, motion mechanisms, and prospective applications in the creation of large machine tools. Consequently, modal, structural, and simulation verification tests were performed using finite element analysis, which has a significant number of applications for structural analysis; in general, the inherent properties of the machine tool structure are unaffected by excitation.

## Data Availability

On reasonable request, the associated author will provide the results of the optimized designs and the basic software for this work. For the acquirement of excess data and material contact corresponding via email.
